# An Interfaith Dialogue on the Neglected Tropical Diseases

**DOI:** 10.1371/journal.pntd.0001240

**Published:** 2011-12-20

**Authors:** Peter J. Hotez, Serap Aksoy

**Affiliations:** 1 Sabin Vaccine Institute and Texas Children's Hospital Center for Vaccine Development, Baylor College of Medicine, Houston, Texas, United States of America; 2 National School of Tropical Medicine at Baylor College of Medicine, Houston, Texas, United States of America; 3 Yale School of Public Health, New Haven, Connecticut, United States of America

*Most of the world's neglected tropical diseases occur among populations that adhere to Islam, Catholicism, or Hinduism, an observation that affords opportunities to establish a unique interfaith dialogue and global action.*

The neglected tropical diseases (NTDs) represent a group of 17 high prevalence and chronic parasitic and related infections [1]–[3]. They are the most common infections of the world's poorest people, especially among the “bottom billion”, which refers to the 1.4 billion people who live below the World Bank poverty level [2]. The NTDs also disproportionately occur in the world's low- and middle-income countries (LMICs) of sub-Saharan Africa [4], Asia [5], [6], and Latin America and the Caribbean [7], although they can also affect the poorest people living in wealthy countries [8], especially indigenous populations [7], [9]. Throughout the developing world, the NTDs have been shown to actually cause poverty because of their adverse effects on child development and intellect, pregnancy outcome, and worker productivity [2].

Yet another dimension of the NTDs is their disproportionate impact on populations living in countries dominated by specific religious affiliations. For example, up to 35%–46% of the world's intestinal helminth infections (ascariasis, trichuriasis, and hookworm infection) and schistosomiasis, as well as a significant percentage of the global trachoma, leprosy, lymphatic filariasis (LF), and onchocerciasis, occur in the member nations of the Organisation of the Islamic Conference (OIC) [10]. The OIC represents a diverse array of nations that include large LMICs in Asia such as Bangaldesh, Indonesia, and Pakistan, Middle Eastern countries, and North African countries such as Chad, Mali, Niger, and Sudan, among others [10]. Similarly, the Catholic-majority countries, led by Brazil, Democratic Republic of Congo, Mexico, and Philippines, account for approximately one-quarter of the world's intestinal helminthiases and leprosy, and 14%–16% of the world's cases of schistosomiasis, as well as almost all of the world's cases of Chagas disease [11], [12], while India, a Hindu-majority country, accounts for 12%–17% of the intestinal helminth disease cases in addition to 41% of the global registered leprosy cases and a significant burden of trachoma [6].

Today, the adherents of Islam, Roman Catholicism, and Hinduism comprise roughly 47% of the world's population (Table 1) [13], yet as shown in Table 2, they account for up to 80% of the world's cases of intestinal helminth infections and 85% of the world's registered leprosy cases, in addition to almost two-thirds of the global cases of schistosomiasis and one-quarter of the trachoma cases. These high prevalence NTDs not only have a devastating health impact on these populations, but as noted above, they also represent major reasons why such populations cannot escape poverty. There is also a curious relationship between the high prevalence NTDs and international conflict [14].

**Table 1:** Global population numbers by religion [13].

Religion	Number of People
Islam	1.3 billion
Roman Catholicism	1.1 billion
Hinduism	0.9 billion
Total	3.3 billion (47%)

**Table 2:** NTDs in the member nations of the Organisation of the Islamic Conference (OIC), Catholic-majority countries, and India.

	Ascariasis	Trichuriasis	Hookworm	Schistosomiasis	LF/Onchocerciasis	Trachoma	Leprosy
**OIC country ** [**10**]	300 million (37%)	243 million (40%)	204 million (35%)	95.5 million (46%)	+	13 million (21%)	43,012 (20%)
**Catholic-majority country ** [**11]**, [12****]	176 million (22%)	168 million (28%)	123 million (21%)	29–34 million (14%–16%)	13 out of 31 countries	1 million (1%–2%)	51,544 (24%)
**India ** [**6**]	140 million (17%)	73 million (12%)	71 million (12%)	-	+/−	1 million (1%–2%)	87,190 cases (41%)
**Total**	616 million	484 million	398 million	124.5–129.5 million	-	15 million	181,746
**% global disease burden**	76%	80%	69%	60%–62%		25%	85%

Through programs of mass drug administration (MDA) it is possible to reduce the prevalence and intensity of many of the most common NTDs, and in the case of LF, onchocerciasis, and trachoma, MDA is leading to the elimination of these diseases as public health problems [15]. Since 2005, efforts have been in place to integrate the elimination or control of the seven most common NTDs, including LF, onchocerciasis, and trachoma, as well as the intestinal helminth infections and schistosomiasis, through a package of drugs administered once or twice yearly and at a cost that averages US$0.50 per person annually [1], [2], [15], [16]. Currently, support for these low-cost packages has been largely limited to the governments of the United States and United Kingdom as well as some private philanthropies [16], [17], but there is an urgent need to expand global support in order to provide MDA for all of the bottom billion at risk for the NTDs. Multi-drug therapy (MDT) for leprosy also represents a low-cost intervention that could lead to the elimination of that disease [15]. For hookworm and schistosomiasis, new vaccines are in development, which might also lead to the elimination of these conditions [18].

The gap in global coverage for MDA and MDT to target the NTDs that disproportionately affect the world's Muslims, Catholics, and Hindus affords an opportunity to establish a unique interfaith dialogue among religious leaders. Given that the NTDs are trapping such populations and faiths in a vicious cycle of poverty and despair, and the extremely low costs of MDA and MDT interventions, religious leaders should be brought together for identifying financial and other mechanisms for ensuring the poorest people gain access to essential medicines for NTDs. Expanding MDA and MDT coverage for NTDs represents one of the lowest cost and cost-effective means to improve health, decrease poverty, and possibly even reduce global conflict in LMICs. A Global Network for NTDs is in place to provide advocacy and to facilitate global intervention [16]. Simultaneously, many of the world's Islamic and Catholic universities could participate in programs of research and development to develop improved control tools in order to effect the elimination of all 17 NTDs. A global summit of religious leaders, academicians at the major religion-affiliated universities, and heads of states of the LMICs would represent an important first step towards these goals.

On behalf of *PLoS Neglected Tropical Diseases*, we want to take this opportunity to thank all of you in the NTD community for your commitment and hard work in 2011. May 2012 bring Peace and Health to everyone!
